# Gel/Space Ratio Evolution in Ternary Composite System Consisting of Portland Cement, Silica Fume, and Fly Ash

**DOI:** 10.3390/ma10010059

**Published:** 2017-01-11

**Authors:** Mengxue Wu, Chen Li, Wu Yao

**Affiliations:** Key Laboratory of Advanced Civil Engineering Materials, Ministry of Education, Tongji University, Shanghai 201804, China; wmx_129@163.com (M.W.); lichen_0712@163.com (C.L.)

**Keywords:** ternary composite system, compressive strength, reaction degree, gel/space ratio, porosity

## Abstract

In cement-based pastes, the relationship between the complex phase assemblage and mechanical properties is usually described by the “gel/space ratio” descriptor. The gel/space ratio is defined as the volume ratio of the gel to the available space in the composite system, and it has been widely studied in the cement unary system. This work determines the gel/space ratio in the cement-silica fume-fly ash ternary system (C-SF-FA system) by measuring the reaction degrees of the cement, SF, and FA. The effects that the supplementary cementitious material (SCM) replacements exert on the evolution of the gel/space ratio are discussed both theoretically and practically. The relationship between the gel/space ratio and compressive strength is then explored, and the relationship disparities for different mix proportions are analyzed in detail. The results demonstrate that the SCM replacements promote the gel/space ratio evolution only when the SCM reaction degree is higher than a certain value, which is calculated and defined as the critical reaction degree (CRD). The effects of the SCM replacements can be predicted based on the CRD, and the theological predictions agree with the test results quite well. At low gel/space ratios, disparities in the relationship between the gel/space ratio and the compressive strength are caused by porosity, which has also been studied in cement unary systems. The ratio of cement-produced gel to SCM-produced gel ($G_{C}$ to $G_{SCM}$ ratio) is introduced for use in analyzing high gel/space ratios, in which it plays a major role in creating relationship disparities.

## 1. Introduction

The use of supplementary cementitious materials (SCMs) in blended cement is considered an effective means to utilize the by-products of industrial manufacturing processes. Furthermore, it significantly reduces the CO_2_ emissions of cementitious materials. Studies of cement-SCM composite systems began by investigating macroscopic properties such as strength, workability, and durability. Subsequently, numerous methods were proposed to determine the reaction degree of cement and SCMs [1,2,3,4,5], and to observe the morphology and transformation of hydration products [6,7,8,9,10]. Researchers have made significant progress toward revealing the evolution of microscopic composites and structures of cement-SCM composite systems. Building on existing testing methods, results, and theories, it is now possible to gain more detailed insight into the influence of the microscopic evolution on the macroscopic properties. Therefore, models that can represent the relationship between the microscopic evolution and the macroscopic properties have received tremendous attention.

Among the macroscopic properties of cement-SCM composite systems, compressive strength has attracted the most attention. This property changes continuously with the hydration of the cement clinkers and the formation of the hydration products. Therefore, the cement reaction degree serves as a simple parameter to describe the microscopic evolutions, and it can predict macroscopic changes, especially in compressive strength [11,12,13]. In addition to the cement reaction degree, the initial volume fractions of water, cement, and air have also been found to affect the compressive strength. As a result, initial porosity has been introduced as a supplement to the relationship between the cement reaction degree and the compressive strength [14]. Odler et al. [15] confirmed the significant effect of initial porosity, finding that the cement reaction degree corresponds to the compressive strength only when the initial porosity for different pastes equals. Subsequent studies further analyzed the relationship between the porosity and the compressive strength [16,17], but the correspondence cannot be perfectly fitted among cement pastes with different mix proportions [18].

Powers et al. [19,20] hypothesized that the compressive strength depends on both the cement hydration and the pore characteristics in the paste. He proposed the concept of the gel/space ratio, defined as the degree to which the gel (hydration products) fills the available space. A typical relationship was then identified between the gel/space ratio and the compressive strength, making it possible to connect the microscopic evolution and the macroscopic properties of cement pastes. Later research investigated this relationship in cement unary systems [19,20,21]. In binary systems containing cement and certain SCMs, this relationship can be also determined by mathematical fitting [22,23,24]. Based on an extended model of the gel/space ratio, further research explored how the nature of hydrates impact the mechanical properties of cement paste [25,26].

Although the gel/space ratio has been studied extensively, neither gel/space ratio evolutions nor the relationship between the gel/space ratio and compressive strength have been elucidated so clearly in binary systems. For ternary composite systems, studies are even scarcer. This work uses the reaction degrees of cement, silica fume (SF), and fly ash (FA) to calculate the gel/space ratio in a cement-silica fume-fly ash ternary system (C-SF-FA system). The effects that the SCM replacements have on the gel/space evolution are analyzed both theoretically and practically on the basis of the SCM reaction degrees. Afterwards, the relationship between the gel/space ratio and the compressive strength is fitted using the exponent model. Finally, the main factors causing the relationship disparities between different samples are discussed.

## 2. Experimental Procedure

### 2.1. Materials

In order to avoid the potential mineral additives in a commercial cement, and to avoid possible errors in the selective dissolution tests, the cement used in this study was prepared by mixing 5 wt % gypsum with cement clinker provided by Conch Cement Co., Ltd. (Wuhu, China) The cement was ground in a ball mill until 80 μm sieve residue constituted less than 1%. SF and FA were commercially available, and the quartz was an analytical-grade chemical reagent. The chemical compositions of the cement clinker, SF, and FA are presented in Table 1.

### 2.2. Sample Preparation

**Figure 1:**
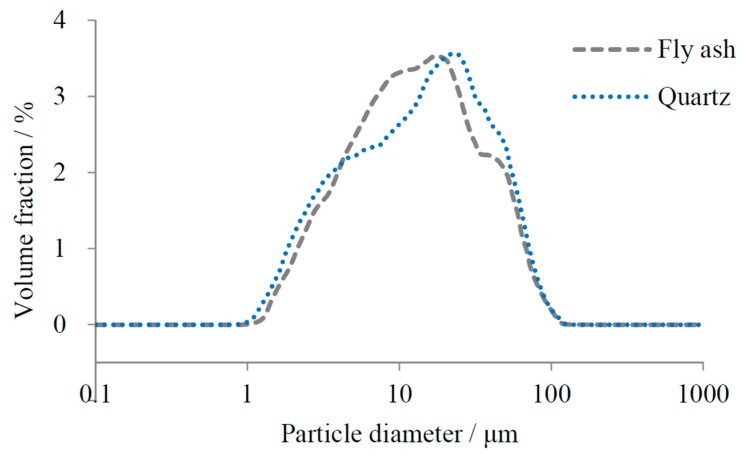
Particle size distribution of FA and quartz.

Cement paste samples were prepared at a constant water/binder ratio of 0.3, and the mix proportions are shown in Table 2. This study used quartz with a similar particle size distribution as a replacement for FA in the C-SF-FA systems, in order to simulate the reaction environment for cement and SF. These samples were designed for auxiliary calculation of the reaction degrees of SF and FA in sample C-1SF-2FA and sample C-1SF-4FA, which will be further described in Section 2.4. The specific surface areas of quartz and FA were 315 m^2^/kg and 312 m^2^/kg, respectively, and their particle size distributions are illustrated in Figure 1.

Specimens were cast in 2 cm × 2 cm × 2 cm steel molds for compressive strength tests, and in Φ 2 cm × 2 cm column plastic molds for reaction degree determination. After 24 h, the specimens were unmolded and hermetically cured at 20 ± 1 °C and relative humidity (RH) ≥95%.

### 2.3. Compression Test

Compressive strength was tested at the curing ages of 1, 3, 7, 28, and 90 d, at a loading rate of 2.4 kN/s. For each sample, six paste specimens were tested for each curing age, and the compressive strengths were averaged.

### 2.4. Determination of SF and FA Reaction Degrees

Image analysis, differential scanning calorimetry, isothermal calorimetry, and selective dissolution are common methods for determining the reaction degree of SCMs in cement composite pastes [4]. However, in the ternary system, it is challenging to determine the reaction degrees of FA and SF separately using the methods mentioned above, because of their similar compositions and dissolution characteristics. In studies by Kocaba et al. [4] and Lothenbach et al. [2], quartz was applied to replace SCMs with low reactivity in order to simulate a similar hydration environment for cement. In this study, we built a parallel cement-silica fume-quartz system (C-SF-Q system) by replacing FA with equal mass of quartz. Quartz is inert, and it provides a similar reaction environment for SF and cement. The reaction degrees of cement and SF in the C-SF-FA system are thought to be the same as those in the C-SF-Q system. Since quartz is insoluble, the SF reaction degree in the C-SF-FA system can be measured on the basis of the C-SF-Q system. By subtracting the SF reaction degree in the C-SF-Q system from the total reaction degree in the C-SF-FA system, which includes both SF and FA, the reaction degree of FA can be isolated.

The SF reaction degree in the C-SF-Q system and the total reaction degree in the C-SF-FA system were determined by selective dissolution. The principle of this method is to dissolve the unhydrated cement as well as the reaction products of cement and SCMs in a certain solution, leaving the unreacted SCMs undissolved. By quantifying the unreacted SCMs, the reaction degree of the SCMs can be determined [27]. In this experiment, EDTA selective dissolution was used based on a protocol outline by Dyson et al. [28]. For each sample, six specimens were tested, and the reaction degrees were averaged.

In fact, FA usually has both physical and chemical effects on cement hydration. Replacing FA with quartz can only simulate the physical effect. However, the chemical effect is usually much weaker than the physical effect at early hydration ages [2,29]. References [4,29] demonstrated that the chemical effect of ground granulated blast furnace slag is so weak that it can be ignored, and by using the quartz replacement method, the physical effect of slag can be separated from the slag hydration. The results also proved that quartz can be used to replace slag and simulate its effects. As for FA, its pozzolanic activity is much lower than that of slag. Before 28 d age, the hydration heat and chemical shrinkage of the paste is nearly the same before and after quartz replacement, according to Reference [29]. Therefore, the physical effects of FA are believed to be much more important than the chemical effects in the quartz replacement method.

**Figure 2:**
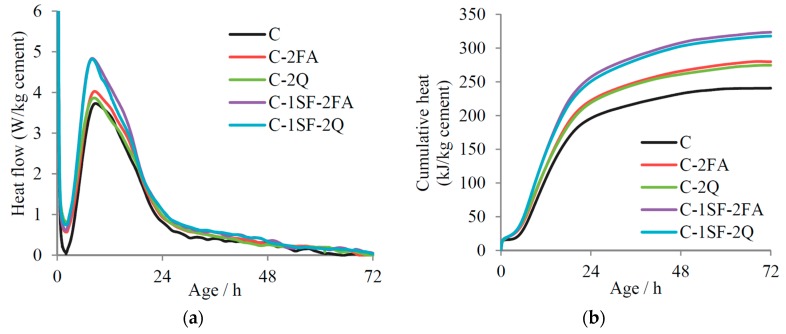
Early-age hydration heat before and after quartz replacement: (**a**) Heat flow; and (**b**) Cumulative heat.

To verify that quartz can well simulate the physical effects of FA, we contrasted the early-age hydration heats before and after quartz replacement, according to the processes in References [4,29]. These tests were conducted by a type SETARAM C80 isothermal calorimetry, and the mix proportions were C-2FA and C-1SF-2FA, representing the binary and ternary systems, respectively. The test temperature was kept at 20 °C, the same as the samples’ curing temperature, and the mass of the binders were about 0.5 g. The test results are illustrated in Figure 2. It should be noted that the heat flow and the cumulative heat in Figure 2 have all been calculated by one kilogram of cement. Usually, FA is almost inert at early hydration ages, which is similar to quartz. If the physical effects of quartz and FA were assumed to be similar, the hydration heat of the samples before and after quartz replacement should be nearly the same. The results in Figure 2 have confirmed this assumption: when the FA was replaced by quartz, the hydration heat remained almost unchanged. The tiny increase of the cumulative hydration heat after quartz replacement could be caused by the quick hydration of the glassy alumina phase in the FA [30].

Based on the references and test results mentioned above, we can conclude that the chemical effects of FA are quite weak at early hydration ages, and the physical effects of FA are similar to those of quartz. Therefore, quartz is believed to produce a similar hydration environment for both cement and SF.

### 2.5. Determination of Cement Reaction Degree

Commonly used methods for determining cement reaction degrees include quantitative X-ray diffraction analysis (QXRD), magic angle spinning nuclear magnetic resonance spectra (MAS NMR), and the non-evaporable water method. The drawbacks in these methods are usually considered to be as follows.

In composite cement systems blended with SCMs, both the SCMs and the hydration products provide a large amount of amorphous material, while the crystal phase content is fairly low [31]. In QXRD, the cement hydration degree is calculated based on the low-content crystal phases, so accuracy is limited in these circumstances [2]. In MAS NMR, overlapping of the peaks of calcium silicate hydrate gel (C-S-H) and SCMs causes deconvolution problems; furthermore, the ferrum in cement and FA leads to line broadening, which generally hinders the use of MAS NMR [32,33]. The application of the non-evaporable water method depends on assumptions about a specific water content bonded by the cement clinker, which is thought to be about 0.23. However, this content has been reported to be variable in different hydrate phases, and the stoichiometry of the hydration reactions vary among different systems [4].

Since all of the methods described have drawbacks, in our previous research, all three methods were tried, and the results suggest that the non-evaporable water method is the most appropriate way to determine cement reaction degrees in discussions of the gel/space ratio [34].

However, it is still uncertain whether the hydration of SCMs creates additional non-evaporable water. Usually, the hydration of the silica phase in SCMs is believed to create no additional non-evaporable water [1,35]; therefore, SF hydration does not make a difference to the non-evaporable water content [36]. Moreover, references [35,37] conclude that the alumina phase released from FA is mostly used to increase the Al/Ca ratio of the C-S-H, during which process the non-evaporable water does not change. This point of view is supported by references [38,39], which demonstrate that the non-evaporable water created by FA was as little as 0.05. References [34,40] conducted some parallel experiments based on QXRD and thermal analysis, which also prove that the non-evaporable water is not sensitive to FA hydration, because FA hydration is quite weak at early ages, and the non-evaporable water created by FA is quite small.

In this work, the non-evaporable water test and calculations of cement hydration degrees were carried out according to the procedures outlined by Schwarz et al. [38], which correct the stoichiometric deviations of the hydration reactions. The ultimate non-evaporable water contents for the cement and FA were believed to be 0.227 and 0.05, respectively [38], while the content for SF was considered to be 0, as discussed above. For each sample, six specimens were tested, and the reaction degrees were averaged.

## 3. Results

The test results presented in this paper are largely in accordance with previous knowledge, and they are therefore not discussed in detail. The main purpose of this work is to analyze the relationships among these results, which will be explored in Section 4.

### 3.1. Compressive Strength

Results for compressive strength are listed in Table 3. The data show that at a small dosage rate (10%), SF improved the strength at all curing ages. In contrast, FA dosing had a negative effect on strength before 7 d, but it increased the strength at more advanced ages. Larger amounts of FA had negative effects, with the compressive strengths of samples C-4FA and C-1SF-4FA being much lower than those of samples C-2FA and C-1SF-2FA, respectively.

### 3.2. Reaction Degrees of SF, FA, and Cement

**Figure 3:**
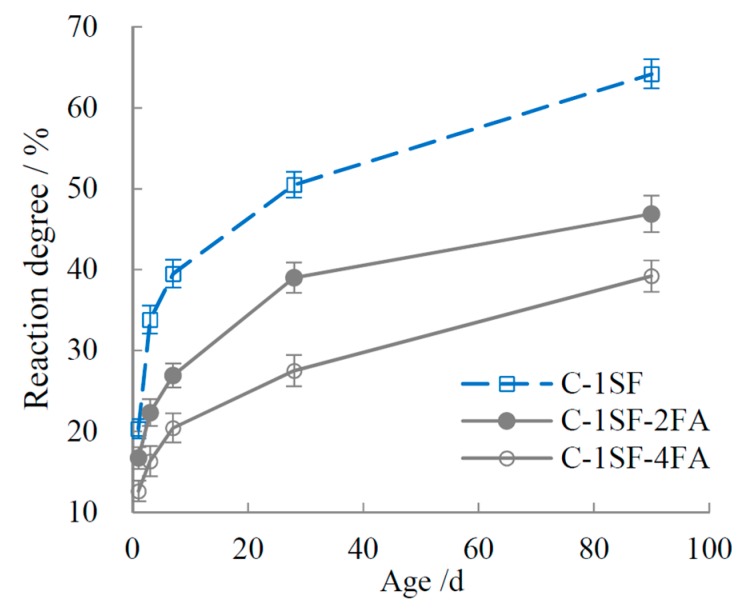
Reaction degree of SF.

**Figure 4:**
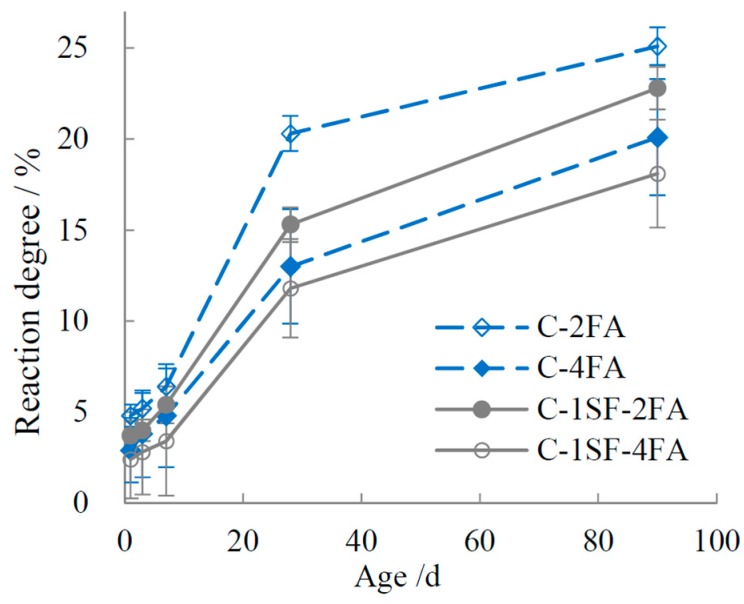
Reaction degree of FA.

The reaction degrees of SF and FA are plotted in Figure 3 and Figure 4. The data indicate that SF showed a higher reaction activity than FA in the composite systems. SF had a high reaction degree before 7 d, but it slowed down later in the process. In contrast, the reaction degree of FA was quite low before 3 d. Taking experimental error into account, we can infer that little FA reacted before 3 d. The reaction of FA started between 3 d and 7 d, and it increased visibly after 7 d. However, owing to its low pozzolanic activity, the FA reaction degrees were much lower overall than those of SF. The evolution of the reaction degrees of SF and FA in ternary systems accords with those in the binary systems. Once mixed in ternary systems, SF and FA show inhibiting effects on one another’s hydration, such that the reaction degrees are lower than those in the binary systems.

**Figure 5:**
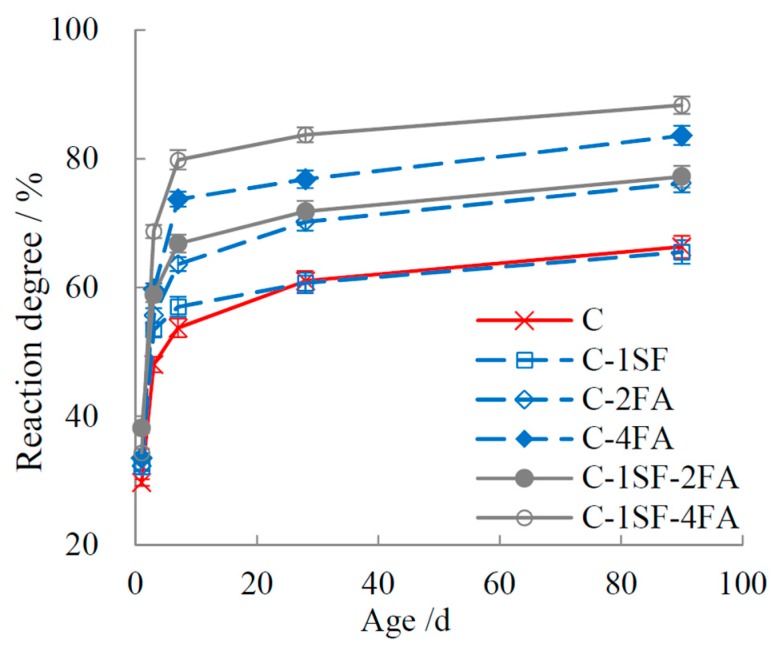
Reaction degree of cement.

Figure 5 outlines the hydration degrees of cement. Due to the dilution effect and nucleation effect of the SCMs [2,41], the hydration degree of cement rose as the dosage of SCMs increased. A comparison of sample C-SF and sample C suggests that before 7 d, SF accelerated the hydration of cement. However, the ingredients with high pozzolanic activity in SF were quickly consumed, such that the cement hydration degree later in sample C-1SF was close to that in sample C. Similar results were found in samples C-2FA and C-1SF-2FA, but the results differed in samples with large dosages of FA. For samples C-4FA and C-1SF-4FA, where the reaction of SF was blocked by the low alkali concentration in the pore solution, the unreacted SF provided a dilution effect to cement at later ages, similar to FA. Consequently, the cement reaction degrees of these two samples stayed high from the beginning to the end.

### 3.3. Gel/Space Ratio

Powers et al. [19,20] described the gel/space ratio in hardened cement paste using Equation (1). Later, Lothenbach et al. [2] introduced a modified model (Equation (2)) to calculate the gel/space ratio in binary cement-fly ash systems.

(1) $$X_{C}=\frac{v_{SG}\alpha_{C}}{v_{C}\alpha_{C}+w/b}$$

(2) $$X_{C-SCM}=\frac{v_{SG}\alpha_{C}m_{C}+2.5v_{SCM}\alpha_{SCM}m_{SCM}}{v_{C}\alpha_{C}m_{C}+v_{SCM}\alpha_{SCM}m_{SCM}+w/b}$$

where *X_C_* and *X_C-SCM_* are the gel/space ratios in unary and binary systems, respectively; $v_{C}$, $v_{SG}$, and $v_{SCM}$ are the specific volumes of cement, gel, and SCMs, respectively; $v_{C}$ = 0.32, $v_{SG}$ = 0.67, $v_{SF}$ = 0.46, and $v_{FA}$ = 0.43, according to the literature [40]; $\alpha_{C}$ and $\alpha_{SCM}$ are the reaction degrees of the cement and SCMs, respectively; $m_{C}$ and $m_{SCM}$ are the mass fractions in the binders of the cement and SCMs, respectively; w/b is the water to binder ratio; and the parameter 2.5 in Equation (2) indicates that the reaction of 1 cm^3^ SCMs occupies 2.5 cm^3^ spaces [22]. The subscript *SCM* stands for *SF* and *FA*.

This research proposes a further modification of the model for use in the C-SF-FA system, as written in Equation (3):

(3) $$X_{C-SF-FA}=\frac{v_{SG}\alpha_{C}m_{C}+2.5v_{SF}\alpha_{SF}m_{SF}+2.5v_{FA}\alpha_{FA}m_{FA}}{v_{C}\alpha_{C}m_{C}+v_{SF}\alpha_{SF}m_{SF}+v_{FA}\alpha_{FA}m_{FA}+w/b}$$

where $X_{C-SF-FA}$ is the gel/space ratio in the C-SF-FA system. Based on Equations (1)–(3), the calculated results for the gel/space ratios of the samples are shown in Table 4.

## 4. Discussion

### 4.1. Relationship between Reaction Degree and Gel/Space Ratio

Based on Equation (3), we can determine how the hydration of cement, SF, and FA theoretically influences the gel/space ratio in the C-SF-FA system. Test results can further verify the theoretical predictions.

It is evident that a higher reaction degree for a certain binder induces an enhanced gel/space ratio. In this section, we analyze whether the evolution of the gel/space ratio is promoted or inhibited when the cement is partly replaced by SCMs. This should depend on the reaction degrees of the cement and the SCMs, as shown in Equation (3).

#### 4.1.1. Binary Systems

In order to determine the effects of SCM replacements in the C-SF-FA system, their effects on binary systems provide valuable foundational knowledge.

**Figure 6:**
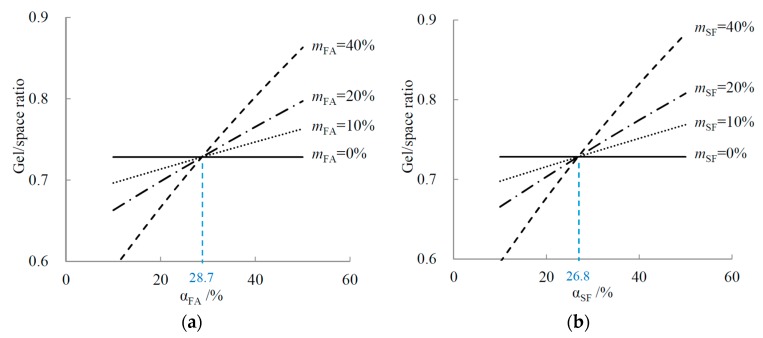
Relationship between gel/space ratio and reaction degree in binary systems: (**a**) FA; and (**b**) SF.

In a cement-FA binary system, the cement reaction degree is initially assumed to be 50%, to make the results more understandable. Based on Equation (2), the relationship between the gel/space ratios and the FA reaction degrees for different FA mass fractions can be simulated, and are illustrated in Figure 6a. The data show that the gel/space ratio rises with increasing FA reaction degree, and all the curves intersect at one point. We define the intersection point as the critical reaction degree (CRD). Figure 6a reveals the different effects that FA replacement exerts on the gel/space ratio, depending on the FA reaction degree: When the FA reaction degree is higher than the CRD, FA replacement has a promoting effect on the gel/space ratio, but when it is lower than the CRD, an inhibiting effect takes place. As the FA mass fraction increases, both the promoting and inhibiting effects are enhanced.

Figure 6b shows the simulated relationship for the cement-SF binary system. The results are similar to those in Figure 6a, except for a different CRD value.

Although the specific value of the CRD is independent of the mass fractions of FA or SF, it is highly dependent on the cement reaction degree. Under the former assumption of $\alpha_{C}$ = 50%, the CRD values are 28.7% and 26.8% for FA and SF, respectively. When the cement reaction degree changes, the CRDs can be mathematically calculated based on Equation (2), as shown in Equations (4) and (5).

(4) $$\alpha_{FA,\;cr}=\frac{0.201\alpha_{C}}{0.056\alpha_{C}+0.322}$$

(5) $$\alpha_{SF,\;cr}=\frac{0.201\alpha_{C}}{0.060\alpha_{C}+0.345}$$

where$\alpha_{FA,\;cr}$ and $\alpha_{SF,\;cr}$ are the CRDs of FA, and SF, respectively.

**Figure 7:**
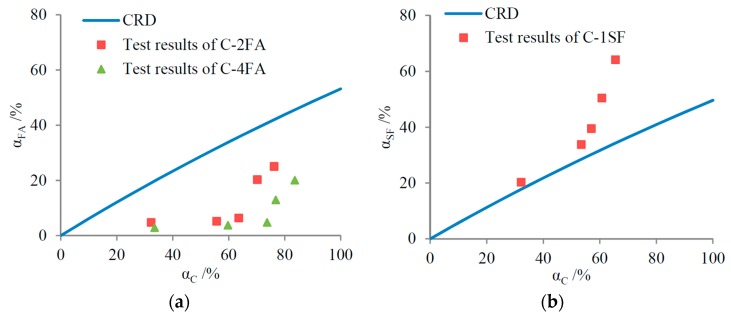
Predictions of effects of SCM replacements in binary systems: (**a**) FA; and (**b**) SF.

Based on the equations above, the SCM replacements’ effect on the gel/space evolution can be predicted by comparing the test results to the CRD, as shown in Figure 7.

As shown in Figure 7, the FA reaction degrees are usually lower than the CRD, demonstrating that the FA replacement inhibits the evolution of the gel/space ratio. In contrast, the SF replacement promotes the gel/space evolution. These results also accord with the different levels of pozzolanic activity between FA and SF. Furthermore, for a higher FA mass fraction, the hydration of cement produces less Ca(OH)_2_, and the hydration of FA is even slower [42]. This explains why the test results for sample C-4FA lie below those of sample C-2FA in Figure 7a, indicating that the gel/space ratio evolution is further inhibited.

In practical binary systems, the gel/space ratio evolution is more complicated. On the one hand, SCM replacements influence the gel/space ratio through pozzolanic reactions, as discussed above; on the other hand, SCMs expedite the hydration of cement [42], which usually contributes to the increase of the gel/space ratio. When the gel/space ratio evolution is considered based on hydration ages, the expedited cement hydration degree must be taken into consideration.

In Table 4, the gel/space ratio of sample C-2FA lags behind that of sample C in the period before 7 d due to the inhibiting effect of FA. Afterwards, the gel/space ratio goes even higher due to the expedited hydration of the cement. Furthermore, the FA mass fraction for sample C-4FA is much larger than that for sample C-2FA, and the enhancement of the gel/space ratio produced by the expedited hydration of cement cannot counter the inhibiting effect produced by the FA replacement. As a result, the development of the gel/space ratio in sample C-4FA is strongly inhibited compared with that of sample C at all curing ages. In terms of SF replacement, the gel/space ratio of sample C-1SF is higher than that of sample C throughout the duration of the experiment. The SF replacement and the expedited cement hydration both contribute to the increased gel/space ratio.

**Figure 8:**
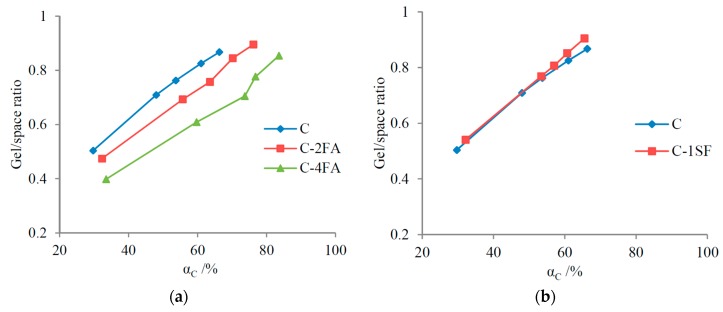
Relationship between gel/space ratio and cement reaction degree in binary systems: (**a**) FA; and (**b**) SF.

The complexity of the practical hydration process does not necessarily lead to the inaccuracy of the theoretical predictions based on the CRDs in Figure 7. As was mentioned above, when the gel/space ratio evolution is predicted by the CRD, equal cement reaction degrees are assumed. However, the test results in Table 4 are based on equal hydration ages. The cement hydration degrees differ when the cement is replaced by SCMs, even if the hydration ages are equal. Combining the results in Table 4 with those in Figure 5, the relationship between the gel/space ratio and the cement reaction degree is displayed in Figure 8. For a given cement reaction degree, the gel/space ratio is definitely inhibited by the FA replacement but promoted by the SF replacement. Contrasting Figure 7 and Figure 8 demonstrates that the predictions for gel/space ratio evolution based on the CRDs are in accordance with the test results.

The analysis above indicates that FA replacement usually inhibits the gel/space ratio evolution, while SF replacement promotes the gel/space ratio evolution in binary systems.

#### 4.1.2. C-SF-FA System

In the C-SF-FA system, the respective effect that FA and SF replacements have on the gel/space ratio is discussed in this section.

**Figure 9:**
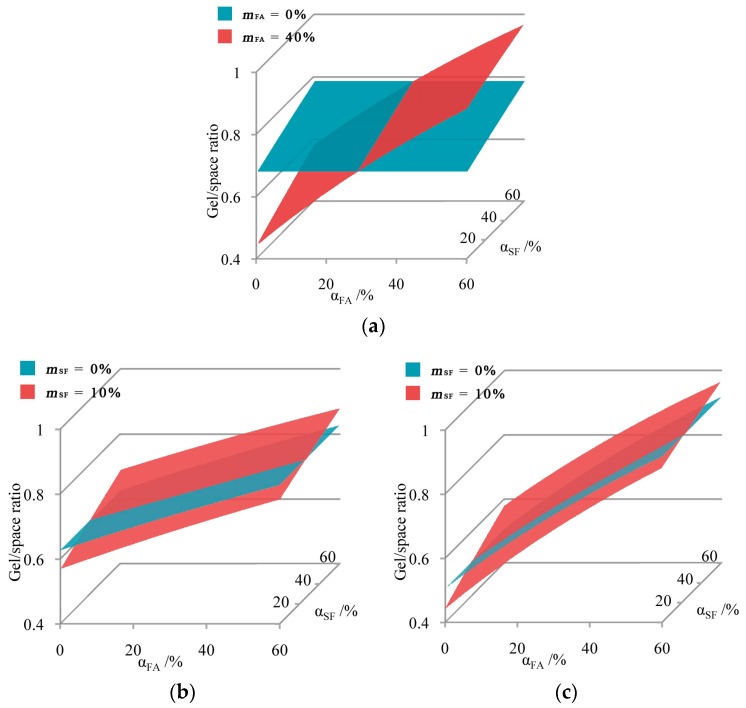
Relationship between gel/space ratio and reaction degrees in the C-SF-FA system: (**a**) FA replacement, when *m_SF_* = 10%; (**b**) SF replacement, when *m_FA_* = 20%; and (**c**) SF replacement, when *m_FA_* = 40%.

To determine the effect of FA replacement, the mass fraction of SF is fixed at 10% according to the experimental designs. Like in the situations in binary systems, the cement reaction degree is assumed to be 50%, to make the results understandable. Based on Equation (3), the relationships among the gel/space ratio, the FA reaction degree, and the SF reaction degree can be simulated, and expressed in Figure 9a. Unlike the situation in binary systems, in which relationships are expressed as curves, the relationship in the C-SF-FA system is expressed as a surface. Moreover, the intersection point in the binary system becomes an intersection curve, which also represents the CRD. Like in the binary systems, when the FA reaction degree is higher than the CRD, FA replacement has a promoting effect on the gel/space ratio, and when it is lower, FA replacement has an inhibiting effect.

To determine the effect of SF replacement, the mass fraction of FA is fixed at 20% or 40%, according to the experimental design. The results are similar to that applied with FA replacement, although the CRDs are different.

In the three situations described above, one FA replacement scenario and two SF replacement scenarios, the CRDs can be mathematically calculated based on Equation (3). These calculations are shown in Equations (6)–(8).

(6) $$\alpha_{FA,\;cr10}=\frac{\left(0.201-0.006\alpha_{SF}\right)\alpha_{C}}{0.051\alpha_{C}+0.322}$$

(7) $$\alpha_{SF,\;cr20}=\frac{\left(0.201-0.011\alpha_{FA}\right)\alpha_{C}}{0.069\alpha_{C}+0.345}$$

(8) $$\alpha_{SF,\;cr40}=\frac{\left(0.201-0.023\alpha_{FA}\right)\alpha_{C}}{0.036\alpha_{C}+0.345}$$

where $\alpha_{FA,\;cr10}$ is the CRD of FA when the SF mass fraction is 10%; and $\alpha_{SF,\;cr20}$ and $\alpha_{SF,\;cr40}$ are the CRDs of SF when the FA mass fractions are 20% and 40%, respectively.

In Equation (6), the value of $0.006\alpha_{SF}$ is far smaller than 0.201. Therefore, it can be ignored, and Equation (6) can be transformed into Equation (9). Similar transformations are also conducted to yield Equations (10) and (11).

(9) $$\alpha_{FA,\;cr10}=\frac{0.201\alpha_{C}}{0.051\alpha_{C}+0.322}$$

(10) $$\alpha_{SF,\;cr20}=\frac{0.201\alpha_{C}}{0.069\alpha_{C}+0.345}$$

(11) $$\alpha_{SF,\;cr40}=\frac{0.201\alpha_{C}}{0.036\alpha_{C}+0.345}$$

**Figure 10:**
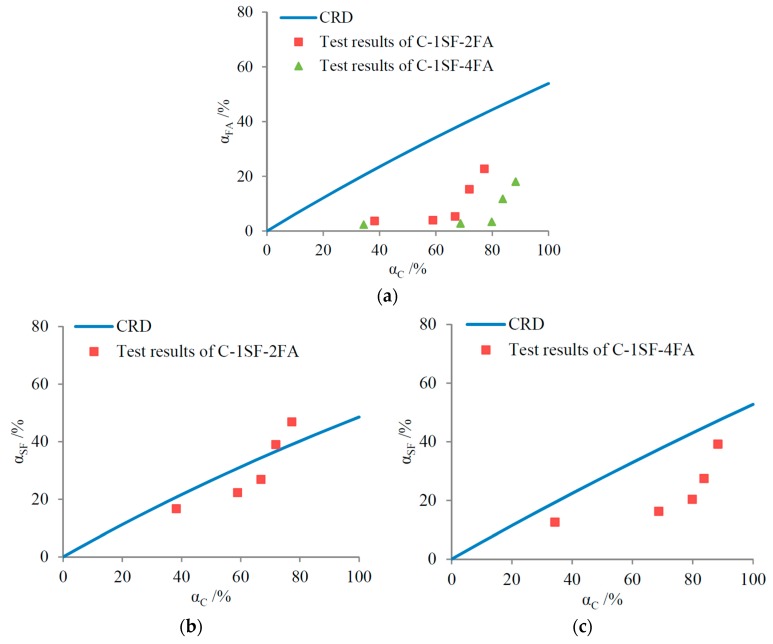
Predictions of effect of SCM replacements in C-SF-FA system: (**a**) FA replacement, when $m_{SF}$ = 10%; (**b**) SF replacement, when $m_{FA}$ = 20%; and (**c**) SF replacement, when $m_{FA}$ = 40%.

These results are surprisingly similar to those in binary systems in which the CRDs depend simply on the cement hydration degree. By comparing the test results to the CRDs, predictions of the promoting or inhibiting effects can be made, as shown in Figure 10.

In the C-SF-FA system, FA replacement inhibits the gel/space evolution, similar to the case in binary systems. However, the SF reaction degrees are mostly lower than the CRDs, especially when the FA mass fraction is 40%. Therefore, SF replacement creates inhibiting effects, which is quite different from the case in the binary systems.

In fact, the cement mass fraction in the C-SF-FA system is much lower than that in the cement-SF binary system. Therefore, cement hydration produces less Ca(OH)_2_. Furthermore, the hydration of FA consumes Ca(OH)_2_, like the hydration of SF does [42,43]. Therefore, SF hydration is restrained in the C-SF-FA system due to the dosing of FA, and the SF reaction degrees become lower than the CRDs. This demonstrates the complexity of the C-SF-FA system, in which the hydration processes of different SCMs also interact.

**Figure 11:**
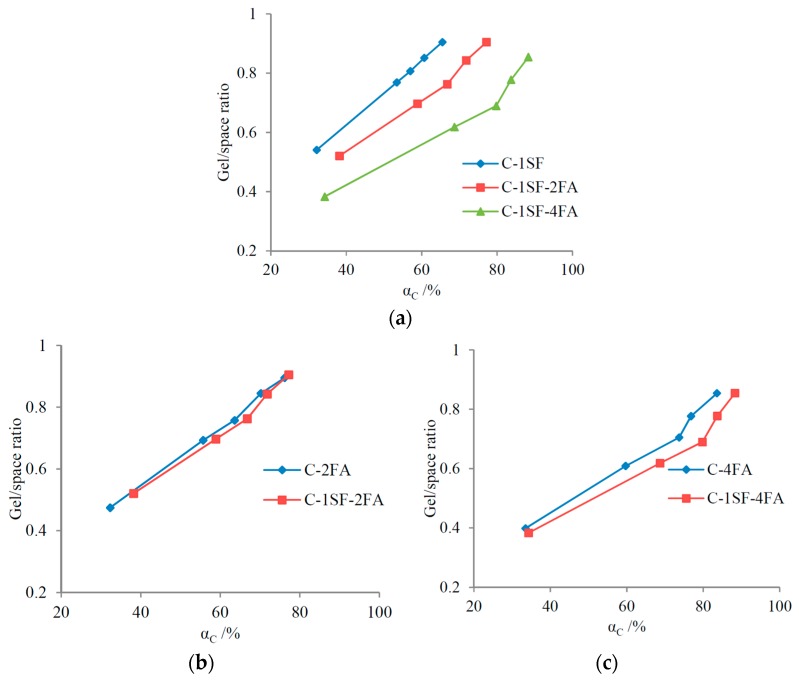
Relation between gel/space ratio and cement reaction degree in the C-SF-FA system: (**a**) FA replacement, when $m_{SF}$ = 10%; (**b**) SF replacement, when $m_{FA}$ = 20%; and (**c**) SF replacement, when $m_{FA}$ = 40%.

In the practical C-SF-FA system, the inhibiting effect of FA replacement is quite evident when comparing the gel/space ratios of samples C-1SF-2FA and C-1SF-4FA to those of sample C-1SF in Table 4. However, comparing the gel/space ratios of sample C-1SF-2FA to those of sample C-2FA suggests that SF replacement mostly has a promoting effect, which is quite different from the CRD predictions in Figure 10b. This may also be caused by the expedited hydration of cement, as was mentioned in the discussion of binary systems. When the gel/space ratio evolutions are compared based on equal cement reaction degrees, the results are shown in Figure 11. Comparing the results in Figure 10 and Figure 11 reveals that the CRD is quite effective in predicting the effects of SCM replacements in the C-SF-FA system.

In conclusion to this discussion of the results in binary and ternary systems, the effects that SCM replacements exert on the gel/space ratio evolution depend on the reaction degrees. Only when the SCM reaction degrees reach a certain proportion of the cement reaction degree can the SCM replacements promote gel/space evolution; the required proportion is indicated by the CRD. From another perspective, this can be also be explained by the different gel volumes created by the binders’ hydration. The hydration of SCMs creates greater volume of gel than that of cement, as is shown in Equations (2) and (3). Therefore, the SCM replacements promote gel/space evolution provided the reaction degrees exceed the CRD. This phenomenon demonstrates the different hydration characteristics of the cement and the SCMs.

### 4.2. Relationship between Gel/Space Ratio and Compressive Strength

To study the relationship between the hydration process and compressive strength, Powers et al. [19,20] built an exponent model fitting the gel/space ratio and the compressive strength, shown here as Equation (12).

(12) $$y=A\cdot e^{Bx}$$

where *A* and *B* are fitting parameters; *y* stands for the compressive strength; and *x* stands for the gel/space ratio (in previous sections, written as *X_C_*, *X_C-SCM_*, and *X_C-SF-FA_* in the unary, binary, and ternary system, respectively).

**Figure 12:**
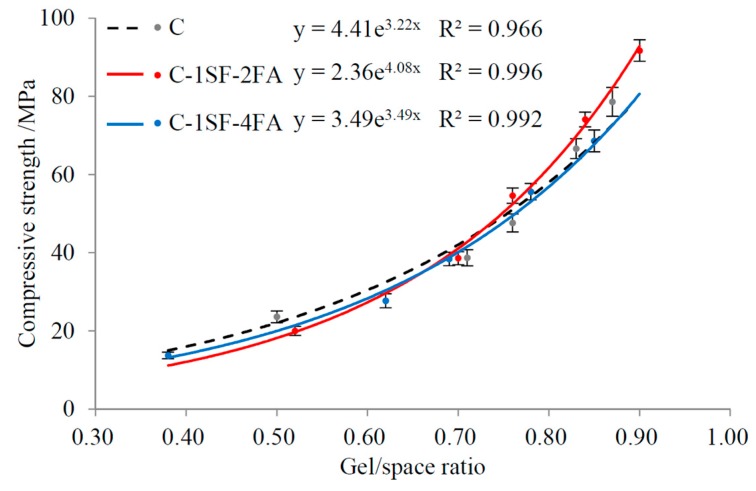
Relation between compressive strength and gel/space ratio.

This model has been widely used in subsequent research [22,44]. This study applies the same model to the C-SF-FA system to discuss the relationship between the gel/space ratio and compressive strength, and the fitting curves are presented in Figure 12. The data show that in the C-SF-FA system, the gel/space ratio fits well with the compressive strength through this exponent relation. However, some disparities exist among the fitting curves of the samples. In this section, we examine the main factors that produce these disparities in the C-SF-FA system.

#### 4.2.1. Influence of Porosity

Previous research based on cement unary systems indicates that the water to binder ratio determines the porosity, which in turn influences the relationship between the gel/space ratio and the compressive strength [26]. In cement unary systems, the porosity is theoretically calculated as Equation (13) [40].

(13) $$P_{C}=\frac{V_{SYS}-V_{SG}-V_{UN}}{V_{SYS}}=\frac{w/b+v_{C}\alpha_{C}-\nu_{SG}\alpha_{C}}{w/b+v_{C}}$$

where $V_{SYS}$, $V_{SG}$, and $V_{UN}$ represent the volume of the whole system, the gel, and the unhydrated clinkers, respectively; and *P_C_* is the porosity of the cement unary system.

**Figure 13:**
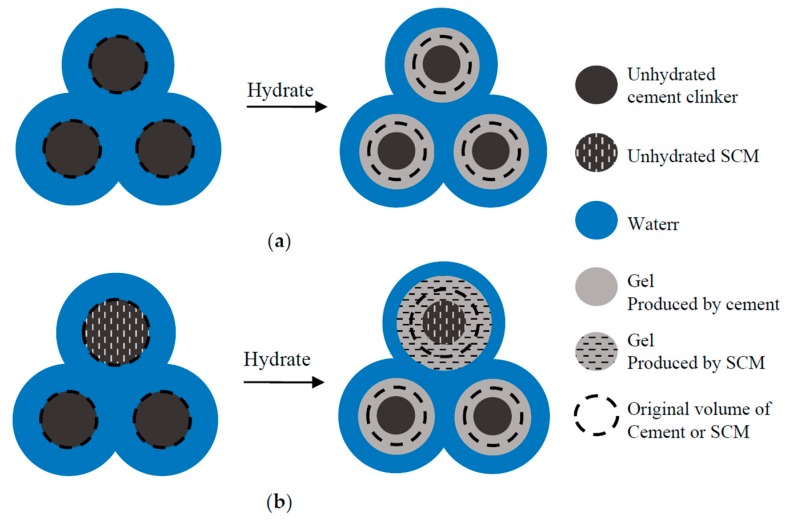
Hydration process of cement paste within the framework of gel/space ratio: (**a**) Cement unary system; and (**b**) C-SF-FA system.

On one hand, within the framework of the gel/space ratio, the hydrated cement pastes consist of unhydrated particles (including cement clinkers and SCMs), gel (including all the hydration products), and the remaining water (including the pores after evaporation), as shown in Figure 13. The gel/space ratio provides the ratio of the gel to both the gel and the remaining water, not considering the proportion of the unhydrated particles. However, the unhydrated particles also have some influence on the compressive strength. Although the compressive strength is mostly determined by the gel/space ratio, it is still influenced by the proportion of unhydrated particles.

On the other hand, porosity provides the ratio of the remaining water to all of the paste. In combination with the gel/space ratio, this can indicate the proportion of unhydrated particles. Therefore, the relationship between the gel/space ratio and the compressive strength is believed to be influenced by porosity. Furthermore, when the water to binder ratio is given in a cement unary system, the volume proportion of the unhydrated particles can also be identified, such that the water to binder ratio has the same effect as the porosity.

Combining Equation (13) with Equation (1), the relationship between the gel/space ratio and the porosity can be written as Equation (14). Clearly, for a certain gel/space ratio, porosity corresponds only to the water to binder ratio, which has the same effect as porosity on the relationship between the gel/space ratio and the compressive strength.

(14) $$P_{C}=\frac{1-X_{C}}{\left(1-\frac{v_{C}}{v_{SG}}X_{C}\right)\cdot \left(1+\frac{v_{C}}{w/b}\right)}$$

In the C-SF-FA system, the porosity can be calculated using Equation (15).

(15) $$P_{C-SF-FA}=\frac{V_{SYS}-V_{SG}-V_{UN}}{V_{SYS}}=\frac{w/b+v_{C}\alpha_{C}m_{C}+v_{SF}\alpha_{SF}m_{SF}+v_{FA}\alpha_{FA}m_{FA}-v_{SG}\alpha_{C}m_{C}-2.5v_{SF}\alpha_{SF}m_{SF}-2.5v_{FA}\alpha_{FA}m_{FA}}{w/b+v_{C}m_{C}+v_{SF}f_{SF}+v_{FA}m_{FA}}$$

where $P_{C-SF-FA}$ is the theoretical porosity of the C-SF-FA system. Combined with Equation (3), the relationship between the gel/space ratio and the porosity can be written as Equation (16).

(16) $$P_{C-SF-FA}=\frac{\left[w/b\cdot \nu_{SG}+(v_{SF}\alpha_{SF}m_{SF}+v_{FA}\alpha_{FA}m_{FA}\right)\left(\nu_{SG}-2.5v_{C}\right)]\left(1-X_{C-SF-FA}\right)}{\left(w/b+v_{C}m_{C}+v_{SF}m_{SF}+v_{FA}m_{FA}\right)\left(\nu_{SG}-\nu_{C}X_{C-SF-FA}\right)}$$

Similar to the situation in the cement unary system, the proportion of unhydrated particles can also be determined based on the combination of the gel/space ratio and the porosity. However, the specific volumes (or densities) of the unhydrated cement clinkers and the unhydrated SCMs are not actually the same in the C-SF-FA system, as shown in Figure 13; neither are the volumes of the gels produced by the cement and the SCMs. The porosity differs at a certain gel/space ratio even when the water to binder ratio is equal. This is shown in Equation (16), where the porosity depends on the reaction degrees and the mass fractions of the binders. Therefore, the porosity can influence the relationship between the gel/space ratio and the compressive strength for two samples, even at the same water to binder ratio.

**Figure 14:**
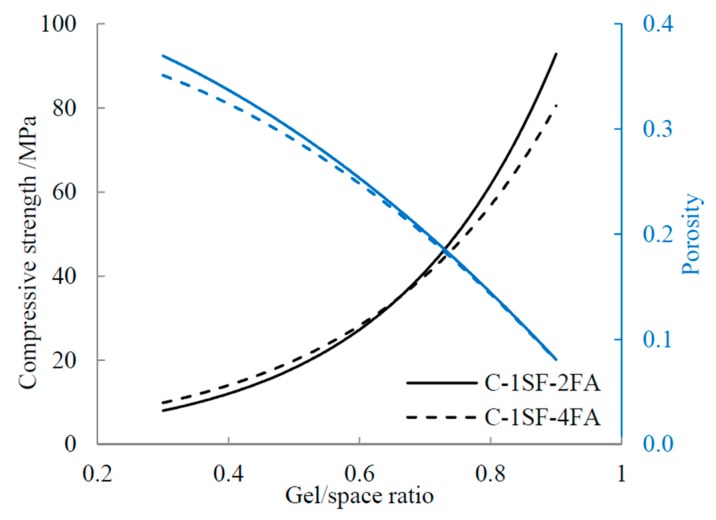
Influence of porosity on gel/space ratio.

The disparities in the relationship fitting curves between samples C-1SF-2FA and C-1SF-4FA can be explained based on the porosity. The porosities of samples C-1SF-2FA and C-1SF-4FA at different gel/space ratios are illustrated in Figure 14.

The curves in Figure 14 suggest that the porosity can explain some of the relationship between the gel/space ratio and the compressive strength when the gel/space ratio is lower than about 0.65. The data indicate that at equal gel/space ratios, lower porosity corresponds to higher compressive strength, which agrees with existing results [45,46]. The two porosity curves gradually overlap at higher gel/space ratios, and the disparity in compressive strength enlarges. This phenomenon demonstrates that some other factor causes the relationship disparities.

#### 4.2.2. Influence of Ratio of Cement-Produced Gel to SCM-Produced Gel

As described in the previous section, the proportion of unhydrated particles can be determined using the porosity. However, this cannot describe all of the complexity in the C-SF-FA system. The unhydrated particles consist of both unhydrated cement clinkers and unhydrated SCMs, and the gel consists of one portion produced by cement and another portion produced by SCM, as shown in Figure 13. The different compositions of unhydrated particles and gel may also influence the compressive strength at certain gel/space ratios. This point of view is supported by the work of Termkhajornkit et al. [25], who emphasized that the nature of the hydrates impacts mechanical properties in the gel/space ratio model. Since the intrinsic strengths of the unhydrated binder particles are usually much higher than those of the gel [47,48,49], the gel is usually considered to be the weakest part of the paste. Therefore, the composition of the gel is believed to influence the compressive strength more than the composition of the unhydrated particles.

**Figure 15:**
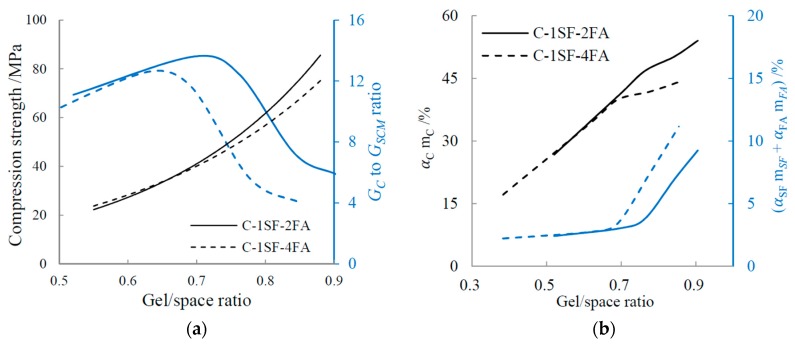
Influence of $G_{C}$ to $G_{SCM}$ ratio on gel/space ratio: (**a**) $G_{C}$ to $G_{SCM}$ ratio; and (**b**) Composition of gel.

As shown in Equation (3), the reaction of 1 cm^3^ of SCMs occupies 2.5 cm^3^ of space, while the reaction of 1 cm^3^ of cement clinkers occupies 2.09 cm^3^ of space. This allows the gel to be divided into cement-produced gel and SCM-produced gel. Therefore, the ratio of cement-produced gel to SCM-produced gel ($G_{C}$ to $G_{SCM}$ ratio) can be used to characterize the composition of the gel. The $G_{C}$ to $G_{SCM}$ ratio is expressed in Equation (17).

(17) $$R_{C/SCM}=\frac{\alpha_{C}m_{C}}{\alpha_{SF}m_{SF}+\alpha_{FA}m_{FA}}$$

where $R_{C/SCM}$ represents the $G_{C}$ to $G_{SCM}$ ratio. The $G_{C}$ to $G_{SCM}$ ratios of samples C-1SF-2FA and C-1SF-4FA at different gel/space ratios are illustrated in Figure 15.

When the gel/space ratio is lower than about 0.65, the $G_{C}$ to $G_{SCM}$ ratios of the two samples are nearly equal, revealing that the gels have similar composition. However, when the gel/space ratio is higher than 0.65, sample C-1SF-2FA has much more cement-produced gel than sample C-1SF-4FA, and the compressive strength of sample C-1SF-2FA is higher. These results indicate that cement-produced gel contributes more to compressive strength than SCM-produced gel for a given gel/space ratio.

In fact, in the framework of the gel/space ratio, the density of cement-produced gel is much higher than that of SCM-produced gel, as shown in Equation (3). For a given gel/space ratio, a higher $\alpha_{C}m_{C}$ value indicates a higher average gel density. Constantinides et al. [47] posited that the high-density gel in the paste has better mechanical properties. Therefore, higher compressive strength can be expected for sample C-1SF-2FA. Furthermore, compressive strength depends mainly on the gel/space ratio, though it is also influenced by the $G_{C}$ to $G_{SCM}$ ratio. Therefore, the decreased $G_{C}$ to $G_{SCM}$ ratios of both samples at high gel/space ratios do not directly decrease the compressive strength.

Based on the discussion above, we can conclude that disparities in the relationship between the gel/space ratio and compressive strength in the C-SF-FA system are dependent on two factors: (a) At early hydration ages, when the gel/space ratio is low, the relationship disparities are influenced by porosity. When the gel/space ratio is equal, the sample with lower porosity shows higher compressive strength; (b) When the gel/space ratio becomes higher as the hydration process goes on, the $G_{C}$ to $G_{SCM}$ ratio becomes relevant as another factor influencing the relationship disparities. For a given gel/space ratio, the sample with a higher $G_{C}$ to $G_{SCM}$ ratio can be expected to have enhanced compressive strength.

## 5. Conclusions

The objective of this work was to establish a link between the microscopic hydration process and macroscopic compressive strength for the C-SF-FA system. By measuring the reaction degrees of the cement, SF, and FA, the gel/space ratios in the C-SF-FA system were determined. Then, the relationship between the gel/space ratio and the SCM reaction degrees was further explored, as was the relationship between the gel/space ratio and the compressive strength. The results can be summarized as follows:

According to theoretical analysis, SCM replacements have two different effects on the evolution of the gel/space ratio, depending on their reaction degrees. When the SCM reaction degrees are higher than the CRD, the SCM replacements show a promoting effect; in contrast, when the SCM reaction degrees are lower, they show an inhibiting effect. These effects become more evident at higher SCM mass fractions. In the practical binary system, FA demonstrates an inhibiting effect due to its low pozzolanic activity, while SF has a promoting effect because of its high pozzolanic activity. In the practical C-SF-FA system, SF displays an inhibiting effect because its hydration process is highly restrained by that of FA.

The gel/space ratio in the C-SF-FA system coincides well with the compressive strength in the exponent relationship. However, the fitting curves between different mix proportions show some disparities. At low gel/space ratios, these relationship disparities are believed to be caused by the porosity, which helps to identify the composition of the paste by revealing the proportion of unhydrated particles. At high gel/space ratios, the $G_{C}$ to $G_{SCM}$ ratio can reveal the detailed composition of the gel, and it plays a decisive role in the relationship disparities.

## Figures and Tables

**Table 1:** Chemical compositions of binders.

Binders	Chemical Compositions (wt %)										
	Na_2_O	MgO	Al_2_O_3_	SiO_2_	P_2_O_5_	SO_3_	K_2_O	CaO	MnO	Fe_2_O_3_	LOI
Cement clinker	0.61	0.92	5.48	23.1	0.07	0.48	0.76	64.8	0.08	3.15	0.55
SF	0.37	0.70	0.30	94.5	0.13	0.40	1.16	0.94	0.02	0.06	1.42
FA	0.45	1.23	28.98	54.70	0.18	0.58	1.65	4.48	0.06	5.24	2.45

**Table 2:** Mix proportions of cement pastes.

Sample No.	Mix Proportions (g)				
	Cement	SF	FA	Quartz	Water
C	100	-	-	-	30
C-1SF	90	10	-	-	30
C-2FA	80	-	20	-	30
C-4FA	60	-	40	-	30
C-1SF-2FA	70	10	20	-	30
C-1SF-2Q	70	10	-	20	30
C-1SF-4FA	60	10	40	-	30
C-1SF-4Q	60	10	-	40	30

**Table 3:** Compressive strength of cement pastes.

Sample No.	Compressive Strength (MPa)				
	1 d	3 d	7 d	28 d	90 d
C	23.6 ± 1.5	38.7 ± 2.1	47.6 ± 2.3	66.6 ± 2.6	78.6 ± 3.7
C-1SF	27.1 ± 1.3	45.6 ± 1.9	57.9 ± 2.0	73.3 ± 2.6	88.5 ± 2.7
C-2FA	22.9 ± 1.0	36.5 ± 1.3	49.4 ± 1.7	70.9 ± 2.2	93.8 ± 2.9
C-4FA	12.8 ± 1.6	31.9 ± 1.8	43.8 ± 2.2	58.0 ± 2.4	71.2 ± 3.1
C-1SF-2FA	20.0 ± 1.2	38.6 ± 1.7	54.6 ± 2.0	74.1 ± 1.9	91.7 ± 2.7
C-1SF-4FA	13.7 ± 0.9	27.7 ± 1.8	38.4 ± 1.7	55.6 ± 2.1	68.6 ± 2.8

**Table 4:** Gel/space ratio of cement pastes.

Sample No.	Gel/Space Ratio				
	1 d	3 d	7 d	28 d	90 d
C	0.50	0.71	0.76	0.83	0.87
C-1SF	0.54	0.77	0.81	0.85	0.90
C-2FA	0.47	0.69	0.76	0.85	0.90
C-4FA	0.40	0.61	0.70	0.78	0.85
C-1SF-2FA	0.52	0.70	0.76	0.84	0.90
C-1SF-4FA	0.38	0.62	0.69	0.78	0.85
